# Black seed oil nanoemulsion containing albendazole against protoscoleces of *Echinococcus granulosus*: An *in vivo* study on C57BL/6 mice

**DOI:** 10.22038/ijbms.2025.83014.17942

**Published:** 2025

**Authors:** Fatemeh Oroojalian, Tahereh Mohammadzadeh, Ailin Ebrahimzadeh, Peiman Alesheikh, Reza Shafiei, Amir Amani

**Affiliations:** 1 Natural Products and Medicinal Plants Research Center North Khorasan University of Medical Sciences, Bojnurd, Iran; 2 Department of Advanced Technologies, School of Medicine, North Khorasan University of Medical Sciences, Bojnurd, Iran; 3 Health Research Center, Life Style Institute, Baqiyatallah University of Medical Sciences, Tehran, Iran; 4 Department of Parasitology and Mycology, School of Medicine, Baqiyatallah University of Medical Sciences, Tehran, Iran; 5 Vector-borne Diseases Research Center North Khorasan University of Medical Sciences, Bojnurd, Iran

**Keywords:** Albendazole, C57BL/6 mice, Echinococcus granulosus, Hydatid cysts, Hydatidosis, Nanoemulsions, Nigella sativa

## Abstract

**Objective(s):**

Hydatid cysts are typically treated with albendazole. Nevertheless, this drug has side effects and limited bioavailability. In this study, we aimed to explore a nanoemulsion of black seed oil to enhance the therapeutic efficacy of albendazole in mice with hydatid cysts.

**Materials and Methods:**

The size of the prepared nanoemulsions was characterized using a Zetasizer analyzer. Additionally, the stability of the nanoemulsions was assessed after 45 days. MTT assay was used to compare the cytotoxicity of free albendazole, nanoemulsion containing albendazole, and nanoemulsion without albendazole. Furthermore, infected mice were treated with these preparations, euthanized, and subjected to autopsy examination. Cysts obtained from mice were examined for histopathological features.

**Results:**

ALB-NE (albendazole-loaded nanoemulsion) DLS results were obtained from black seed oil. Freshly prepared ALB-NE showed (d50 = 170 nm), PDI: 0.323, ALB-NE after 45 days storage at 25 ºC were (d50 = 92.4 nm), and ALB-NE after 45 days storage at 45 ºC revealed (d50 = 118 nm). The cytotoxicity of albendazole was reduced when loaded into the nanoemulsion. Moreover, the group treated with nanoemulsion containing albendazole showed a significant decrease in size and number of cysts compared to those receiving free albendazole or nanoemulsion without the drug. Additionally, after 60 days, the nanoemulsion containing albendazole showed 100% survival, while the survival rate was 50% for free albendazole, 75% for nanoemulsion without albendazole, and 37.5% for PBS.

**Conclusion:**

The nanoemulsion containing albendazole can be a promising treatment for hydatid cysts.

## Introduction

**Figure 1:**
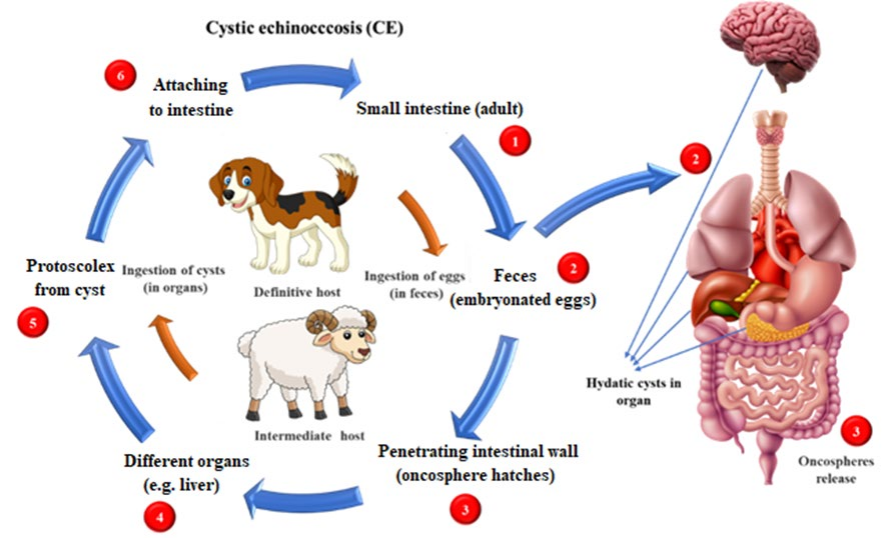
Schematic diagram of the life cycle of *Echinococcus granulosus*

Hydatidosis, also known as cystic echinococcosis, is a parasitic disease caused by the larval stage of the dog tapeworm *Echinococcus granulosus*. Hydatidosis is widespread worldwide and is commonly endemic, where humans are close to domestic animals and canids (1, 2). The infection is transmitted when carnivores (e.g., dogs) excrete parasite eggs in their feces. Humans are infected directly (through contact with the eggs) or indirectly (e.g., ingesting contaminated raw vegetables). As a result, cysts develop in internal body organs (e.g., lungs and liver, see Figure 1) (3, 4). 

Surgical removal of the cysts is the preferred treatment when cysts are large, located in the lungs, or have been infected secondarily. Alternatively, drug therapy is recommended for patients who cannot undergo surgery or have inactive, uncomplicated cysts in the liver (5-7). Broad-spectrum anthelmintics, including albendazole (ALB), mebendazole, praziquantel, and fenbendazole, are typically used for this purpose (8, 9). In particular, ALB, which inhibits the polymerization of tubulin in the larval parasite and reduces cyst size and/or causes parasite death, is a common medication. Treatment with ALB is associated with preventing recurrence and avoiding surgery (10, 11). However, its bioavailability is extremely low (< 5% in humans) (12). Additionally, ALB can cause adverse effects such as abdominal pain, nausea, vomiting, dizziness, diarrhea, liver dysfunction, leukopenia, and hematuria (11).Natural products and compounds have served as herbal remedies since the dawn of human civilization (13, 14). Phytochemicals, secondary metabolites derived from plants, possess various biological activities in humans, including potential anticancer, antibacterial, antifungal, anti-viral, and parasitic effects (15, 16). In contemporary times, a significant number of the leading pharmaceutical products are either natural compounds or their derivatives (17-19). Nanoemulsions are isotropic oil, surfactant, and water mixtures with particle sizes less than or around 100 nm (20, 21). As drug carriers, they offer numerous advantages, for example, easy preparation procedure, feasibility of being produced on a large scale, protection of the drug against hydrolysis and oxidation, improving the absorption efficiency of the drug, minimizing the overall dose required, and reducing the side effects of the drug. In recent years, nanoemulsion-based carriers have received significant attention for delivering water-insoluble compounds in different administration routes, including topical (22, 23), ophthalmic (24), and inhalation (25). In oral applications, nanoemulsions can improve bioavailability, efficiency, and/or pharmacokinetics, as in the case of mebudipine (26), amlodipine (27), felodipine (28), curcumin (29), and atovaquone (30).

Black seed (*Nigella sativa*, Latin name *Niger nigella*) possesses notable pharmaceutical properties, including anticancer, anti-allergic, anti-hypertensive, anti-corruption, antibacterial, anti-parasitic, cardioprotective, and immunostimulatory effects. This plant is also beneficial in treating infections caused by intestinal worms, especially in children (31). This study assessed the effectiveness of ALB-loaded nanoemulsion prepared with black seed oil in treating hydatid cysts in C57BL/6 mice.

## Materials and Methods

Tween 80, Span 80, DMEM, FBS, MTT (3-(4,5-dimethylthiazol-2-yl)-2,5-Diphenyltetrazolium Bromide) and penicillin-streptomycin (10,000 U/ml) were purchased from Sigma-Aldrich (USA). Black seed oil was prepared by cold pressing the seeds.

### Preparation and characterization of black seed oil nanoemulsion

Black seed oil, Tween 80, Span 20, and ethanol were mixed (10 min, 600 rpm) at room temperature (see Table 1- supporting information). Subsequently, water was added dropwise and stirred for 45 min to obtain a clear, viscous colloidal solution. The mixtures were kept for 24 hr (room temperature, away from light). Samples that showed any sign of phase separation, precipitation, or turbidity were discarded. The particle size was determined by dynamic light scattering (DLS, Scatteroscope I, K-ONE.LTD, Korea).

Albendazole-containing nanoemulsion (ALB-NE) was prepared by adding ALB to the mixture of oil, surfactants, and co-surfactants before adding water. 

### Stability of the nanoemulsions

To evaluate the stability of nanoemulsions over time, the particle size of freshly prepared nanoemulsions and those assessed 45 days post-preparation at a temperature of 25 °C was measured using a particle size analyzer. Additionally, the turbidity of the emulsions was examined visually.

### In vitro cytotoxicity evaluation

The cytotoxic effects of the preparations on the L929 cell line were investigated using the MTT colorimetric assay. Mouse Fibroblast L929 Cells were purchased from Mashhad University of Medical Sciences (Mashhad, Iran). Cells were maintained in Dulbecco’s Modified Eagle’s Medium enriched with 10% heat-inactivated fetal bovine serum and penicillin (100 units/ml)/streptomycin (100 μg/ml). The cultured cells were maintained in a humidified 37 °C incubator containing 5% CO_2_. Before each experimentation, cells were starved with DMEM containing 0.5% FBS for 24 hr.

 The cells were grown (24 hr) in 96-well plates (original density 1 × 10^4^ cells/well) in 100 μl DMEM medium having 10% FBS (32, 33). The cells were treated with the preparation, and after 48 hr incubation, 20 μl MTT solution (5 mg/ml) was added to each well for three hr. Afterward, the medium was discarded, and DMSO (150 μl) was added to each well. A plate reader (Tecan Group Ltd. Mannedorf, Switzerland) measured the absorbance at 570 (34, 35).

### Preparation of protoscoleces

The livers of sheep infected with hydatid cysts were collected from industrial slaughterhouses in Shiraz and Tehran (Iran) and transferred to the parasitology lab. After washing the surface of the cysts with 70% ethanol, the whole hydatid fluid containing protoscoleces was drained out of the cyst in sterile conditions and transferred to a 50 ml falcon tube. Protoscoleces were then allowed to precipitate and washed three to five times with sterile normal saline. Microscopically, the viability of protoscoleces was assessed with 0.1% aqueous eosin solution. Unstained (viable) protoscoleces were used for injection into animals.

### Animal studies

Forty-eight male 9-week-old C57BL/6 mice were used for the animal studies (Ethical approval code: IR.NKUMS.REC.1397.107). Twenty microliters of the protoscoleces sediment was diluted to a final volume of 0.3 ml of phosphate buffer saline (PBS) with Penicillin (100 units) and streptomycin (200 mg/ml), and injected into the peritoneal cavity of the animals (36, 37). Eight months after the injection, the mice were gavaged with either of the following treatment groups (1 ml final volume): 

- Free ALB (suspension containing 50 mg/kg) 

- ALB-NE (nanoemulsion containing 50 mg/kg ALB)

- NE (nanoemulsion without ALB)

- Negative control (PBS)

After three months, mice were euthanized, and the cysts were harvested and examined.

### Data analysis

GraphPad Prism 8.0 software was used to analyze the data. Descriptive statistics were presented as mean ± standard deviation (SD). A one-way ANOVA test was used to detect differences between groups. To determine group differences, we used Tukey *post hoc* pairwise comparison. *P*-value<0.05 was used to qualify the significant differences.

## Results

### Characterization of the nanoemulsion

**Figure 2:**
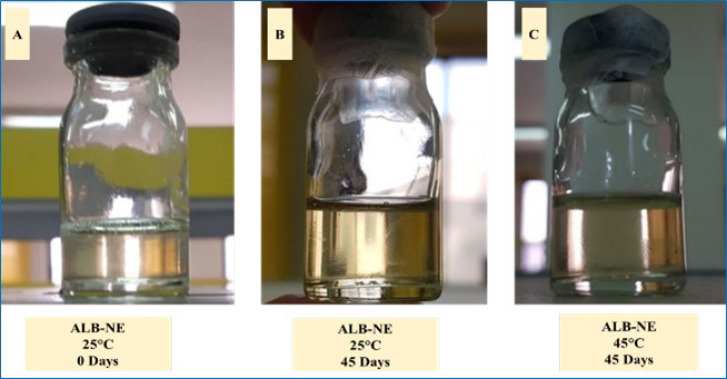
Freshly prepared ALB-NE (albendazole-loaded nanoemulsion) (A) and after remaining at 2525 ℃ and 4545 ℃ for 45 days

**Figure 3:**
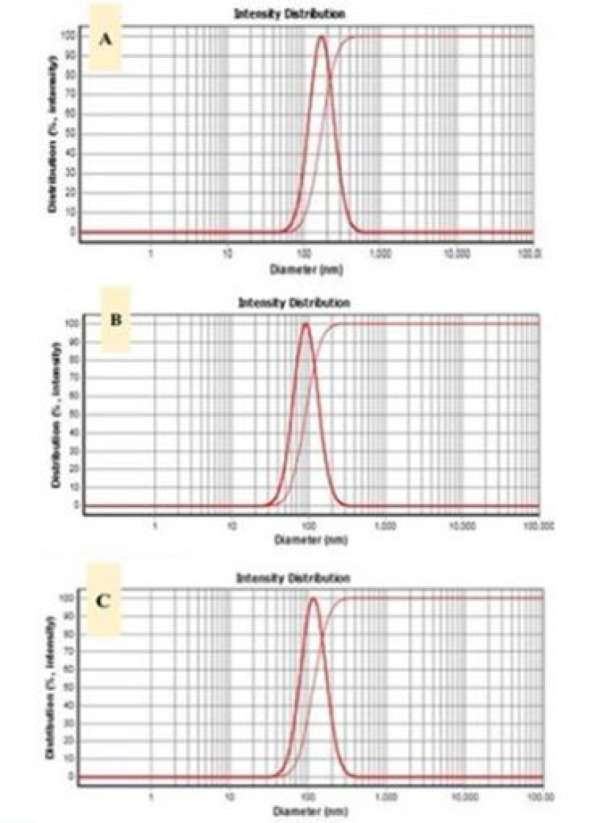
DLS results of ALB-NE (albendazole-loaded nanoemulsion) obtained from black seed oil

Figure 2 shows the appearance of ALB-NE when freshly prepared and stored for 45 days at 25 ºC and 45 ºC. The preparations indicated no sign of separation phase or turbidity. The particle size results for the samples are given in Figure 3. The findings from visual examination and DLS indicate that the nanoemulsion remains stable for a minimum of 45 days at 45 ºC. 

### In vitro cytotoxicity

**Figure 4:**
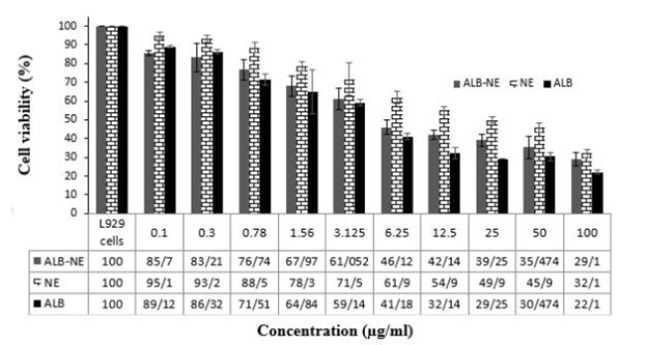
MTT assay results of free ALB, ALB-NE (albendazole-loaded nanoemulsions), and NE (albendazole-free nanoemulsions) on L929 cells after 24 hr

Figure 4 represents the results of the MTT assay of ALB, ALB-NE, and NE. As the details show, ALB appears to be the most cytotoxic preparation (mean±SD IC50 5.24±1.51 µg/ml), while NE has minimum toxicity (mean±SD IC50 20.51±1.12 µg/ml). The cytotoxicity of ALB-NE falls between that of the other two samples (i.e., mean±SD IC50 8.66±1.80 µg/ml).

### In vivo studies

**Figure 5:**
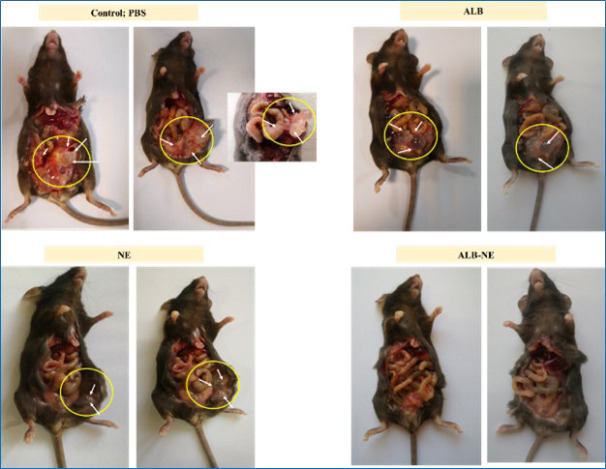
C57BL/6 mice infected with hydatid cysts were assigned to different experimental groups

**Figure 6:**
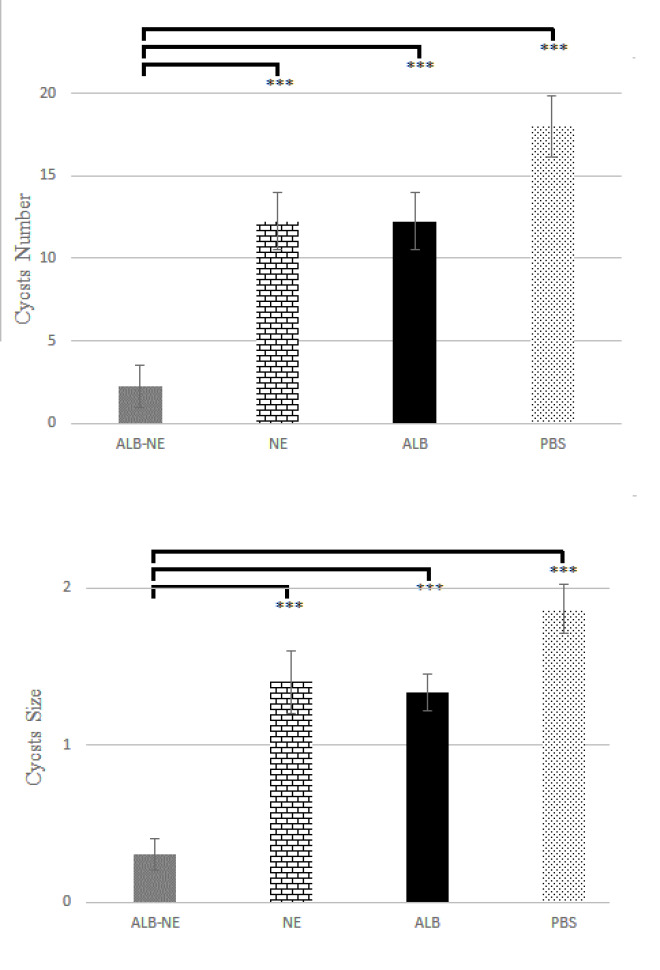
Number (top) and size (bottom) of hydatid cysts in C57BL/6 mice across experimental groups

Figures 5 and 6 depict the results of treatment of the infected mice. The details show that cysts’ size and number decreased significantly in mice receiving ALB-NE, compared with the other three groups (*P*-value<0.5). Mean±SD sizes of cysts were 0.3±0.1, 1.4±0.2, 1.3±0.1, and 1.9±0.2 mm for ALB-NE, NE, ALB, and PBS treatment groups, respectively. Also, the mean±SD numbers of observed cysts were 2.3±1.3, 12.3±1.7, 12.3±1.7, and 18.0±1.8 for ALB-NE, NE, ALB, and PBS treatment groups, respectively. 

**Figure 7:**
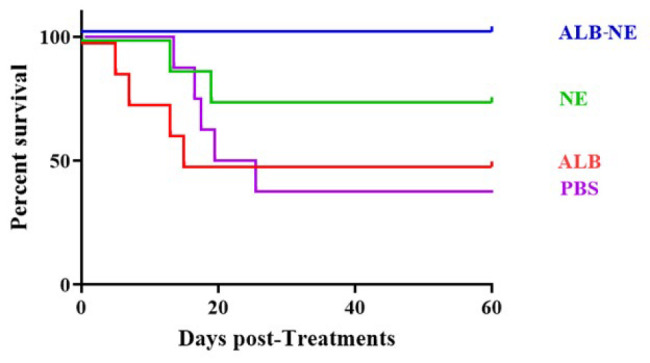
Kaplan-Meier plot (survival diagram) for C57BL/6 mice infected with hydatid cysts across experimental groups

Figure 7 shows the survival diagram for the different treatment groups. After 60 days of treatment, ALB-NE was the only group that showed 100% survival, while other groups indicated 50% (ALB), 75% (NE), and 37.5% (PBS) survival.

*Histological analysis*

**Figure 8:**
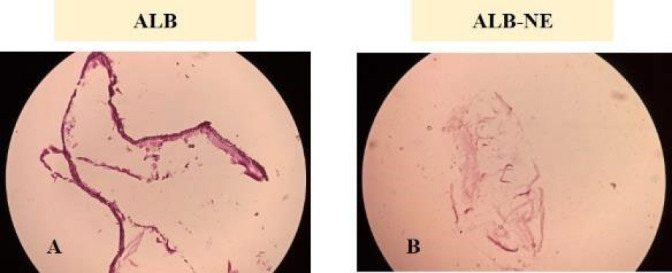
Histopathological findings in the mice receiving free albendazole (ALB) and albendazole-loaded nanoemulsion (ALB-NE)

The pathological microscopic observation showed no protoscolex or brood capsule proliferation from the germinal layer of cysts in ALB or ALB-NE treated groups (see Figure 8). Additionally, the pathological analysis of collected cysts in the ALB-treated group showed typical germinal and laminated structures, which are well-formed layers. In contrast, parasites recovered from ALB-NE treatment exhibited changes in the germinal layer, such as breakage discontinuity and looseness. Furthermore, upon visual examination, the liver damage signs were less prominent in the group receiving ALB-NE compared with that of ALB (free albendazole).

## Discussion

This study highlights the superiority of a black seed oil nanoemulsion as a vehicle in treating hydatidosis. The particle size of the nanoemulsion was below 200 nm. It is well-known that particle size > 200 nm often activates the complement system. Thus, the particles are cleared rapidly from the blood (38). Additionally, obtaining minimum 45-day stability at 45 ºC, while not enough for the market, can still provide sufficient stability for the study period. 

The MTT assay showed free albendazole with an IC50 value of 5.12 µg/ml, comparable with a previous report (i.e., 0.69-8.1 µg/ml, depending on the cell line) (39). The free nanoemulsion (without albendazole) had minimum cytotoxicity, arguably due to its biocompatible ingredients. When the albendazole was loaded into the nanoemulsion, the cytotoxicity increased due to the presence of albendazole as a relatively cytotoxic compound. We also believe that the nanoemulsion particles may shield the toxic albendazole molecules, thereby protecting the cells from direct contact with albendazole. Nonetheless, it is worth noticing that while the MTT assay is a facile and cost-effective approach, it is not ideal for assessing the toxicity of nanoemulsions: By diluting nanoemulsions, they commonly break up. So, the MTT test measures the cytotoxicity of the broken emulsion rather than the nanoemulsion. Therefore, findings from *in vitro* tests (including the MTT test) should be interpreted cautiously as they are associated with sample dilution (20). 

The *in vivo* results show a significant reduction in cyst size and number in the nanoemulsion containing albendazole compared to free albendazole and the nanoemulsion without the drug. Nanoemulsions play a crucial role in enhancing the activity of anti-parasitic agents. In an *in vitro* report, the spiramycin nanoemulsion showed superior activity against Tachyzoites of *Toxoplasma gondii.* Specifically, while the nanoemulsion containing 250 µg/ml spiramycin exhibited > 70% mortality, the suspension of spiramycin with the same concentration had < 10% mortality (40). Nanoemulsion containing atovaquone was also reported to be effective against *T. gondii* *in vitro* and *in vivo*. Increased bioavailability, reduced number and size of brain cysts, and better survival rates were observed in infected mice treated with nanoemulsion containing the drug, compared with the nanoemulsion without the drug (30). Curcumin is loaded in nanoemulsions and mouse models’ acute and chronic phases of toxoplasmosis (29). Teimouri *et al.* showed that the scolicidal effect of curcumin nanoemulsion on the mortality rate of protoscoleces that were exposed to 1250 and 625 µg/ml of curcumin for 60 min, was 94 and 73.33%, respectively (41).

Several mechanisms have been proposed regarding the fascinating effects of nanoemulsions on drug bioavailability and their permeation through biological membranes: they enhance the solubility of the lipophilic drug (42), form lipoproteins (to facilitate lymphatic transport) (43), and modify the properties of the biological barriers (44, 45). Arguably, nanoemulsions increase its permeation through different biological membranes, including cyst walls. Both mechanisms have played roles in enhancing the scolisidal activity of albendazole. Undoubtedly, further work is required to understand the underlying mechanisms better.

A) freshly prepared ALB-NE (d50 = 170 nm), B) ALB-NE after 45 days storage at 25 ºC (d50 = 92.4 nm), C) ALB-NE after 45 days storage at 45 ºC (d50 = 118 nm).

ALB: free albendazole, NE: nanoemulsion without albendazole, ALB-NE: nanoemulsion containing albendazole.

ALB: free albendazole, NE: nanoemulsion without albendazole, ALB-NE: nanoemulsion containing, PBS: control group. *** indicate *P*-value<0.001.

ALB: free albendazole 50 mg/kg, NE: nanoemulsion without albendazole, ALB-NE: nanoemulsion containing 50 mg/kg albendazole and PBS: control group.

## Conclusion

Human echinococcosis is a zoonotic infection resulting from tapeworms belonging to the genus *Echinococcus*. The present study showed significant reductions in both cyst size and number and a lower mortality rate in infected mice treated with albendazole-loaded nanoemulsions compared to those receiving free albendazole. These findings suggest that albendazole-loaded nanoemulsion holds promise as a treatment for hydatidosis, eliminating the need for long-term treatment with free albendazole and reducing its adverse effects.

## Declaration

We have not used any AI tools or technologies to prepare this manuscript.
